# Properties of Bread Dough with Added Fiber Polysaccharides and Phenolic Antioxidants: A Review

**DOI:** 10.1111/j.1750-3841.2010.01815.x

**Published:** 2010-10

**Authors:** Anusooya S Sivam, Dongxiao Sun-Waterhouse, SiewYoung Quek, Conrad O Perera

**Affiliations:** Authors Sivam and Sun-Waterhouse are with The New Zealand Inst. for Plant & Food Research LtdPrivate Bag 92169, Auckland 1025, New ZealandAuthors Sivam, Quek, and Perera are with Food Science, Chemistry Dept., The Univ. of AucklandAuckland, New Zealand. Direct inquiries to author Sun-Waterhouse (E-mail: Dongxiao.Sun-Waterhouse@plantandfood.co.nz)

**Keywords:** bread, dough, fiber polysaccharides, functional food, phenolic antioxidants, wheat protein

## Abstract

During breadmaking, different ingredients are used to ensure the development of a continuous protein network that is essential for bread quality. Interests in incorporating bioactive ingredients such as dietary fiber (DF) and phenolic antioxidants into popular foods such as bread have grown rapidly, due to the increased consumer health awareness. The added bioactive ingredients may or may not promote the protein cross-links. Appropriate cross-links among wheat proteins, fiber polysaccharides, and phenolic antioxidants could be the most critical factor for bread dough enhanced with DF and phenolic antioxidants. Such cross-links may influence the structure and properties of a bread system during baking. This article presents a brief overview of our current knowledge of the fate of the key components (wheat proteins, fibers, and phenolic antioxidants) and how they might interact during bread dough development and baking.

## Introduction

Bread is a staple processed food that dates back over 12000 y. Numerous studies have been devoted to various aspects of breadmaking—a process in which wheat flour, water, salt, sugar, and yeast are mixed in varying proportions into viscoelastic dough subjected to fermentation and baking. Bread is a leavened food produced via fermentation of wheat flour sugars derived from starch involving chemical interactions of various food components in the employed ingredients. These interactions can be adjusted to create desirable products, once the underlying chemical and physical processes are well understood.

Consumer awareness of the importance of functional foods has greatly grown in the past years (Krystallis and others 2008). The global market for functional food is expected to increase to €14.7 billions by 2013 (The Medical News, 2010, http://www.news-medical.net). Functional foods with elevated levels of antioxidants and dietary fibers (DFs) are of high demand because of their associated health benefits, including maintenance of health and protection from diseases, such as cancer, cardiovascular diseases, and degenerative diseases (Boyer and Liu 2004; Pelucchi and others 2004; Arts and Hollman 2005; Scott and others 2008). As bread is a common component in western diet, it may be a convenient food to deliver fiber polysaccharides and phenolic antioxidants of high concentrations. In this article, we review the published literature on experimental and mathematical studies of breadmaking to identify the scopes of further investigation into functional bread, with particular reference to the incorporation of fiber and phenolic ingredients and associated technical challenges. We consider the chemical composition of the common ingredients used in breakmaking, with a specific focus on the impact of added fiber and/or phenolic constituents on dough functionality and bread quality, and with less emphasis on the optimization of bread processing and effects of processing on fortified ingredients. We also discuss the interactions among wheat proteins, fiber polysaccharides, and phenolic antioxidants during dough development and baking process, and the mechanisms associated with the changes in the structure and conformation of wheat proteins, fiber polysaccharides, and phenolic antioxidants.

## Key Wheat Flour Components and Their Functions

### Wheat protein—the “gluten”

Wheat is normally used to make bread, pasta, and noodles, because among the cereal flours, only wheat flour has the ability to form cohesive doughs upon hydration (Létang and others 1999; Landillon and others 2008). Wheat dough entraps gas, which is essential for the production of light and leavened products such as bread and pastry (Peighambardoust and others 2010). These properties are mainly attributed to the gluten proteins that generate a continuous viscoelastic network during dough development (Belton and others 1995; Shewry and others 2001). Protein constitutes only 7% to 15% of common wheat flour including albumins and globulins, and gluten accounts for 80% to 90% of flour proteins (Daniel and Triboi 2002; Shewry and others 2002).

**Figure 1:**
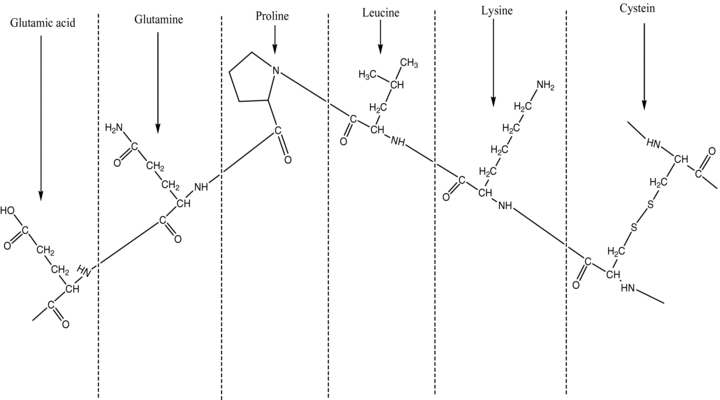
Structure of some common amino acids found in wheat, showing linkages with peptide bonds (from Atwell 2001).

Wheat proteins naturally occur as oligomers of different polypeptides containing more than 35% hydrophobic amino acid residues (isoleucine, leucine, tryptophan, tyrosine, valine, phenylalanine, and proline). There is 6% to 12% proline in wheat proteins. The structure of some amino acids in wheat proteins is shown in Figure 1 (Atwell 2001). The molecular weight of protein generally ranges from thousands to millions, with those of wheat proteins being from 30000 to more than 10 million Daltons (Hoseney 1994; Wieser 2007). Some protein chains are branched polymer (Kent-Jones and Amos 1967; Wieser 2007). The presence of nonrotating N-C bond in the ring structure of proline facilitates a fixed Φ angle of 70° in the protein molecules, resulting in a random or aperiodic structure in the proteins containing higher levels of proline residues (Damodaran 1996; Xu and others 2001).

When dough is washed in running water, the starch is removed and a viscoelastic rubbery mass is obtained that is called “gluten” (Hargreaves and others 1995; Bloksma and Bushuk 1998). Gluten is a protein complex, with proline (10%), glycine (20%), and glutamine (approximately 35%) being the most abundant amino acids responsible for gluten development (Fermin and others 2003; Pommet and others 2005; Wellner and others 2005). Gluten proteins can be categorized based on their solubility into gliadins (alcohol–water soluble) and glutenins (insoluble) (Wieser 2007). Gliadins and glutenins are well known for their influence on the properties of gluten (Lagrain and others 2008). The gliadins create viscosity required for dough development, whereas the glutenins provide strength and elasticity of dough (Toufeili and others 1999).

Not only the structure of gluten protein but also the bonding within the protein plays important roles in the dough development and functionality. Proteins contain covalent and noncovalent bonds that contribute to dough formation and structure (Hoseney 1994; Bushuk 1998). Noncovalent bonds include hydrogen bonding, hydrophobic interactions, ionic bonds, and Van der waals interactions (Hoseney 1994). Although hydrogen bonds are individually weak, they create stability to the dough when large numbers of bonds are established during dough development. Hydrophobic and ionic bonds, although present in very small amounts, play significant roles in the interactions among the biopolymers within bread dough that consequently promote dough stability. On the other hand, covalent bonds (namely peptide bonds and disulphide bonds) exist among the amino acids that normally remain unchanged during breadmaking (Bushuk 1998; Aït Kaddour and others 2008). Although cystein accounts for only 2% of gluten protein, it can significantly influence the structure and functionality of gluten (Wieser 2007). The sulphydral group of a cystein can react with another cystein residue to form a disulphide bond (-S-S-). Two cystein residues, either derived from the same protein via intramolecular bonding or different protein chains via intermolecular bonding, can form a loop within the protein (Hoseney 1994). During dough development, these disulphide bonds can be mobilized through disulphide interchange reactions (Bushuk 1998; Fermin and others 2003; Abang Zaidel and others 2008). When gluten proteins are heated up to 75 °C, sulphydryl-disulphide interchanges are accelerated (Sun and others 2008). Disulphide bonds among flour proteins form strong cross-links within and between polypeptide chains, which stabilizes other energetic bonding such as hydrogen and hydrophobic interactions. Another type of covalent bond form during breadmaking is tryosine–tryosine cross-link between gluten proteins and tryosine-dehydroferulic acid, or between gluten proteins and nonstarch carbohydrates—arabinoxylans from flour (Wieser 2007).

**Figure 2:**
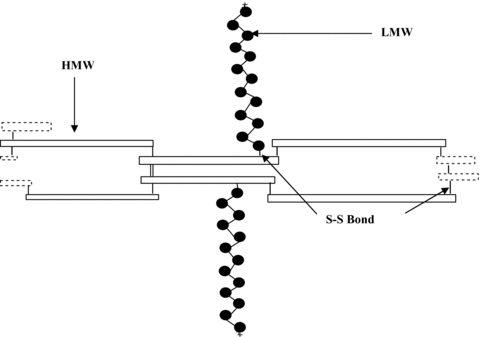
Model unit showing interchain disulphide structures of LMW (•) glutenin subunits and HMW (□) glutenin subunits (adapted from Wieser 2007).

The polymers of glutenins are made up of high molecular weight (HMW) subunits (60 to 90 kDa) and low molecular weight (LMW) subunits (10 to 70 kDa) (Belton and others 1995; Wellner and others 2005; Anjum and others 2007). HMW subunits, accounting for 5% to 10% of total gluten protein, are formed among high molecular mass glutenin polymers (also called “glutenin macro polymers”). They range from 67.5 to 73.5 KDa (approximately 630 to 830 amino acids). These polymers commonly occur in allelic forms (He and others 2005; Pirozi and others 2008) and influence gluten's viscoelasticity. Disulphide bonds significantly stabilize these polymers (Lefebvre and Mahmoudi 2007), functioning as interchain bonds between HMW subunits, and between HMW and LMW subunits (Shewry and others 2001). HMW subunits form an “elastic backbone” of head-tail polymer with interchain disulphide bonds, and the resultant backbone becomes the basis for LMW subunits to attach via disulphide bonds (Shewry and others 2001) (Model HMW and LMW subunits as shown in Figure 2, Wieser 2007). Furthermore, gliadins can also interact with the glutenin polymers via noncovalent hydrophobic interactions and the glutamine residues via hydrogen bonds (Wellner and others 2003). Interchange of disulphide bond occurs during dough mixing, possibly via breaking, and reforming of disulphide bonds (Damodaran 1996; Bloksma and Bushuk 1998). Breaking and reforming disulphide bonds results in the formation of a network aligned along the direction of extension (Shewry and others 2001).

**Figure 3:**
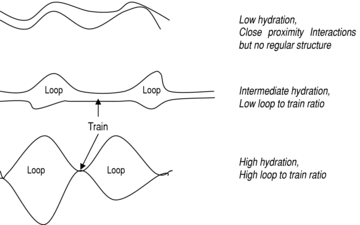
Model for the effect of hydration on the loop to train ratio of HMW subunits (from Belton 1999).

“Loop and train model” was first proposed by Belton (1999) to describe the behavior of gluten upon hydration (Figure 3). Hydrated gluten contains intermolecular β-sheet in addition to α-helix and β-turn structures, indicating the important role of HMW subunits in gluten elasticity (Belton and others 1995; Wellner and others 2005). HMW glutenins comprise (1) extensive and repetitive sequences that can form loose β-reverse turns in solution and subsequently give a β-spiral structure, (2) short and nonrepetitive domain that are rich in α-helix (Shewry and others 2002; Wellner and others 2003, 2005).

At low level of hydration (for example, <35%), most of the protein–protein interactions occur via interchain hydrogen bonding between glutamine residues in the β-spiral structures (Belton and others 1995; Shewry and others 2001, 2002). When hydration level increases, plasticization of a system facilitates the formation of hydrogen-bonded structures between chains, allowing the orientation of the β-turns in adjacent to β-spiral to form “interchain”β-sheet (Shewry and others 2001, 2002). Further increase in hydration to high level leads to the formation of hydrogen bonds between water and glutamine, forming loops where they do not interact with each other (Shewry and others 2001, 2002; Wellner and others 2005). When hydration increases, the amount of train region in the β-sheet conformation is reduced, resulting in more loop regions (Belton 1999). This loop and train model is consistent with the results from a later fourier transform infrared (FTIR) study on protein conformational changes during gluten extension (Wellner and others 2005) and an investigation on the repetitive peptides of various numbers of HMW subunits at different levels of hydration (Feeney and others 2003).

On the other hand, glutenin subunits form a disulphide-bonded network in dough. An extension on dough would result in a strain in the network, which can be described using a stretched loop and “unzipped” train regions (Shewry and others 2001). Water was proposed to have a major role in the loop and train model, and elevated water content leads to an easy-to-deform system (Belton and others 1995; Belton 1999; Shewry and others 2001). Increased train content in the dough system would exhibit an increased resistance to extension. The trains are associated with β-sheets conformation, and the resistance to extension depends on the proportion of HMW subunits in gluten proteins (Belton 1999). Moreover, LMW subunits can make the dough stronger and less extensible (Grassberger and others 2003). In summary, the length and nature of HMW subunits play a vital role in the viscoelastic properties of dough (Belton 1999). The addition of glutenins with HMW and LMW subunits to dough would greatly influence rheological and baking properties. Table 1 lists some published review articles on the gluten structures.

**Table 1:** Reviews on the gluten structures

References	Strength
Belton 1999	Loop and train model development, explaining protein–protein interactions, association of subunits can take place by interchain hydrogen bonding. Many hydrogen bonds that cannot be broken, at the same time, there will be nonbonded mobile region (loops) and bonded regions (trains).
Shewry and others 2001	Novel detail of glutenin subunits and details of their molecular structures and interactions that allow development of model to explain their role in determining the viscoelastic properties.
Shewry and others 2002	HMW subunits of glutenins are important for high level of elasticity (dough strength), these subunits can be manipulated by genetic engineering, leading to either increased dough strength or change the structural properties of gluten.
Autio 2006	Naturally occurring oxidative enzymes in flour or added to dough might oxidise water-extractable arabinoxylans via ferulic acid bridges, resulting arabinoxylan gel that will hinder gluten formation.
Wieser 2007	Different structural domains of gluten due to variability caused by genotype.

### Wheat starch

Wheat starch accounts for 63% to 72% of flour (Hoseney 1994). The building block of starch and glucose can form both linear polysaccharides (called amylose) and branched polysaccharides (called amylopectin) (BeMiller and Huber 1996). The wheat starch commonly used contains approximately 25% amylose and 75% amylopectin (BeMiller and Huber 1996). Wheat starch granules exist in 2 lens sizes: type B (spherical size, 1 to 3 μm diameter) and type A (larger granules, 20 to 45 μm diameter) (Hoseney 1994; Atwell 2001). Interactions between starch granules and gluten are possible, occurring via hydrogen bonding and preventing bread staling (Ottenhof and Farhat 2004).

### Nonstarch polysaccharides

**Figure 4:**
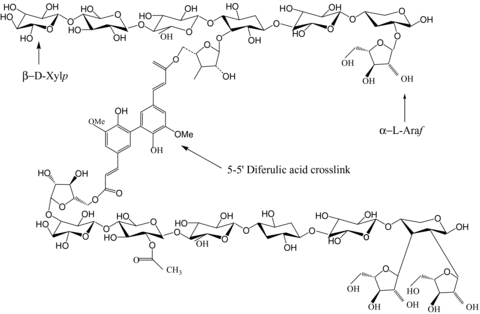
Wheat arabinoxylans: β-(1→4)-D-xylan backbones substituted with single and/or double α-L-arabinofuranoside moieties and covalently cross-linked through a 5,5′ diferuloyl moiety. α-L-Ara*f*=α-L-arabinofuranose, β-D-Xyl*p*=β-D-xylopyranose (from Adams and others 2003).

Other important components of flour are nonstarch fiber polysaccharides that are derived from wheat cell walls. These fiber polysaccharides account for 2% to 4% of flour (Atwell 2001). Both the flour from the whole grains and the flour that has been refined can be used for bread. The former is nutritionally preferable to the latter. Arabinoxylans and mixed 1,3-/1,4- β-D-glucans are 2 major wheat fiber polysaccharides or nonstarch polysaccharides (Biliaderis and others 1995; Sasaki and others 2000). The structure of arabinoxylans varies significantly among types of cereal, containing xylan backbone branched with arabinosyl residues in various linkages (Nandini and Salmath 2001). Arabinoxylans may also be linked to ferulic acids covalently through ester linkages to the arabinose as shown in Figure 4 (Adams and others 2003). The wheat fiber polysaccharides influence the functionality of gluten (Autio 2006). Arabinoxylans significantly influence water balance and rheological properties of bread dough, as well as retrogradation of starch. Arabinoxylans are able to absorb high amounts of water (Brennan and Cleary 2007; Prasad Rao and others 2007), causing reduced water availability for gluten during dough development (Autio 2006). The water-absorbing capacity of arabinoxylans also affects the distribution of moisture among dough constituents, resulting in altered dough rheological properties and prolonged mixing time (Wang and others 2002a). The redistribution of moisture among gluten and other macromolecules allows arabinoxylans to react directly with gluten molecules, generating a more complex network containing both gluten and arabinoxylans (Autio 2006). Other molecules such as arabinogalatctan peptides also interact with gluten, resulting in a reduced water absorption and extensibility (Autio 2006).

### Dietary fiber

Since the endogenous fiber polysaccharide content in wheat flour is only 2% to 4%, the incorporation of DF in bread would raise the health profile of the final bread products (Jenkins and others 1998; Pelucchi and others 2004; Scott and others 2008). Fibers in various forms have been previously used in breadmaking (Anil 2007). The definition of DFs has been debated and has evolved in the past (Trowell and others 1976; Prosky 2000; AFSSA 2002; European Commission 2008). A final agreement was only reached in November 2008 on a global DF definition for the Codex Alimentarius (ALINORM 09/32/26 2009). Now the Codex defines DF as carbohydrate polymers with 10 or more monomeric units, which are not hydrolyzed by the endogenous enzymes in the small intestine of humans. DFs decrease intestinal transit time, increase stool bulk, reduce total and LDL cholesterol level in blood, decrease postprandial blood glucose and insulin level, buffer excessive acid in the stomach, and prevent constipation (Harvey and others 1973; Cummings and others 1978; Burr and others 1985; Bourquin and others 1996; Erkkilaä and others 1999; Peters and others 2003; Jenkins and others 2004; Brennan and Cleary 2007; Lunn and Buttriss 2007). Low DF intake has been associated with health problems such as diverticular disease, diabetes, obesity, coronary heart disease, and colorectal cancer (Kromhout and others 1982; Jenkins and others 1998; Levi and others 2001; Anderson and others 2004; Scheppach and others 2004; Leeds and Benjamin 2005; Jiménez and others 2008; Kendall and others 2010). Various DF daily intake ranges are recommended in different countries, for example, 20 to 25 g in Japan (Mori and others 1996), 20 to 35 g in America (American Dietetic Assn., 2002), 25 to 30 g in Australia and New Zealand (Natl. Health and Medical Research Council and Dept. of Health, http://www.thefreelibrary.com), and 30 to 40 g in France (Bagheri and Debry 1990).

In addition to associated health benefits, incorporation of DF to food products imparts a number of functional properties to the finished foods, including increased water holding, gel forming, stabilizing, texurizing, and thickening capacities (Gelroth and Ranhotra 2001; Kunzek and others 2002; Dikeman and others 2006). DF may stabilize or modify food physical structure and product density because of its fibrous nature (Gelroth and Ranhotra 2001). It was found that purified DFs from orange, pea, cocoa, coffee, wheat, and microcrystalline cellulose had pronounced effects on dough rheological behavior yielding higher water absorption and smaller extensibility than those obtained without fiber addition (Gomez and others 2003). Dry potato pulp Potex and 2 derived enzymatically treated fiber powders containing a high level of lignin and insoluble nonstarch polysaccharides had led to increased hardness, deformation modulus, and gumminess (Kaack and others 2006).

### Phenolic antioxidants

**Figure 5:**
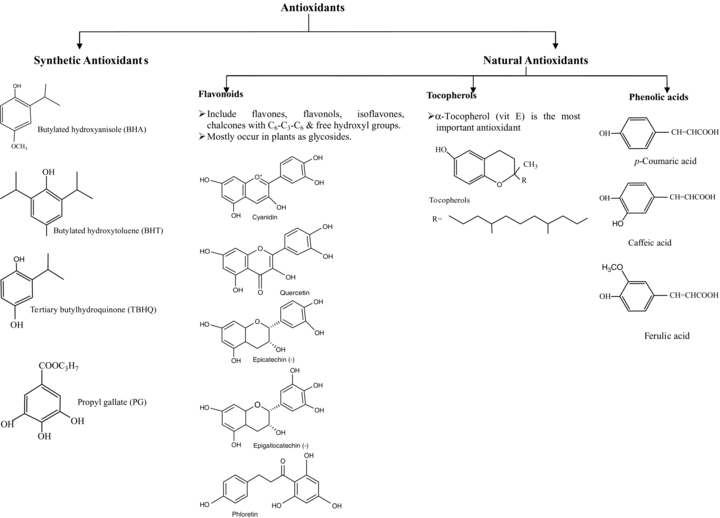
Phenolic antioxidants (from Gordan 2001; Shi 2001; Kyoung Chun and Kim 2004; Thaipong and others 2006; Liyana-Pathirana and Shahidi 2007; Wojdyło and others 2007).

Cereal grains contain intrinsic phenolic antioxidants. These compounds not only scavenge free radicals in the biological systems (Miller 1996; Bilgiçli and others 2007), but also prevent food spoilage (Gordan 2001). Free radicals derived from a wide range of biological reactions in the body can damage essential biomolecules. Excess of unscavenged free radicals cause unhealthy conditions as well as diseases, for example, reactive oxygen species (ROS) including superoxide (O_2_^−^), hydroxyl radical (OH), hydrogen peroxide (H_2_O_2_), and lipid peroxide radicals have been associated with chronic degenerative diseases such as cancer, inflammatory, aging, cardiovascular, and neurodegenerative disease (Shahidi and Naczk 1995; Arts and Hollman 2005). Natural antioxidants such as flavonoids, tocopherols, and phenolic acids (structure as shown in Figure 5) may inhibit lipid peroxidation in food and improve food quality (Gordan 2001; Shi 2001; Kyoung Chun and Kim 2004; Price and others 2006; Thaipong and others 2006; Fan and others 2007; Liyana-Pathirana and Shahidi 2007; Wojdyło and others 2007). The modes of action include direct scavenging (for primary antioxidants, for example, α-tocopherol) and indirect scavenging (for secondary antioxidants, for example, dilauryl thiodipropionate and thiodipropionic acid) (Shahidi and Naczk 1995; Gordan 2001).

Cereal grains contain phenolic acids, saponins, phytoestrogens, and flavonoids (Liyana-Pathirana and Shahidi 2007). Ferulic, vanillic, and *p*-coumaric acids are the most abundant free phenolic acids in wheat exhibiting antioxidant activities (Zielinski and Kozlowska 2000). The antioxidant activity of wheat products depends on the nature of antioxidant species, wheat variety, extraction method, and type of antioxidant activity assay (Fardet and others 2008).

## Changes in Product Properties due to the Interactions among Bread Components

Breadmaking is a complex process mainly consisting of mixing, fermentation, and baking, in which water evaporation, volume expansion, starch gelatinization, protein denaturation, and crust formation occur. Dough mixing transforms the mixture of flour and water into a homogenous viscoelastic dough for subsequent dough development and air occlusions (Haraszi and others 2008; Manu and Prasad Rao 2008). During dough mixing, the mechanical energy imparted induces conformational changes in wheat proteins, namely, breakage and formation of both covalent (-SS-) and noncovalent (hydrophobic and hydrogen) bonds (Aït Kaddour and others 2008). Water must be added to the optimal absorption level so that dough can reach a stage of “optimum development” (Wesley and others 1998; Aït Kaddour and others 2008). Baking is the last but also an important step involving heat and mass transfer, causing physical, chemical, and structural changes of dough components (Sablani and others 1998; Mondal and Datta 2008). Increased temperature would promote the formation of protein cross-links, causing setting of the loaf during baking. Leavening is accomplished through trapping the CO_2_ resulted from yeast fermentation in the dough system (Mondal and Datta 2008). The yeast is inactivated when temperature reaches approximately 54 °C. Starch gelatinization becomes noticeable at 65 °C and further enzymatic action stops at 76 °C (Kent-Jones and Amos 1967). Wheat protein denaturation and starch gelatinization both influence water diffusion by releasing and absorbing water (Singh 2005). Several changes occur simultaneously while baking. Some of the possible reactions and interactions are discussed below.

### Changes in wheat starch

During baking, the 3 hydroxyl groups of the glycosyl residue of wheat starch may form hydrogen bonding with water. The overall hydrogen bonding effect would become significant when starch is heated in water at 52 to 85 °C; crystallinity and birefringence of granules would be lost. This process is called gelatinization (Funami and others 2005). When heating is prolonged, the starch granules break and are eventually disrupted. Amylose and amylopectin are dispersed. This is called pasting. On cooling and aging, the starch molecules can reassociate and form new crystals. This process is called retrogradation (Hoseney 1994; BeMiller and Huber 1996). Starch gelatinization, pasting, and retrogradation are common events in the baking process and are highly associated with rheological behavior (Atwell 2001; Kim and Cornillon 2001). During breadmaking, water moves from hydrated gluten to starch granules causing gelatinization. Interactions between gelatinized starch granules and the gluten network occur in crumb, causing a loss of kinetic energy and subsequently an increase in firmness (Ottenhof and Farhat 2004). Further to the study of BeMiller and Huber (1996) on starch gelatinization using differential scanning colorimetry (DSC), Ritota and others (2008) used nuclear magnetic resonance (NMR) to characterize the dynamic starch-water interaction at heating temperatures ranging from 20 °C to 77 °C, and found that water molecules might have different relaxation rates, depending on their diffusive and chemical exchange with starch components.

### Changes in wheat proteins

During baking, wheat proteins undergo structural changes because of their heat susceptibility (Schofield and others 1983; Hayta and Schofield 2004). The decreased protein content is possibly due to gradual breakdown of protein during fermentation. A decrease in protein content of bread dough during fermentation has been detected by size exclusion-high-performance liquid chromatography (SE-HPLC) (Singh 2005; Maforimbo and others 2006). Polymeric proteins decrease and LMW proteins increase, and there is a reduction in protein solubility caused by aggregation or cross-linkage during baking (Danno and Hoseney 1982). A cleavage of interchain disulphide bonds within glutenins would cause an increased amount of components with reduced molecule size (Gupta and others 1993). Therefore, intact disulphide bonds preserve large polymer structure of glutenin (Maforimbo and others 2006).

During baking, temperature of the crumb starts from about 70 °C and can reach 100 °C within 30 min (Singh 2005). Two thermally induced phenomena related to baking are starch gelatinization and protein denaturation (Kent-Jones and Amos 1967). Gluten proteins are heat susceptible, and their performance or functionality during breadmaking would decrease when heat is applied and would be completely lost at 75 °C (Falcão-Rodrigues and others 2005; Lagrain and others 2005). During dough formation, proteins become hydrated (Feeney and others 2003). When temperature rises during baking, water migrates from gluten to the starch, promoting starch swelling. Increased cross-linking and polymerization of gluten polymers occur during baking, as a result of increased sulphydryl (SH) and disulphide (SS) interchange reactions (Schofield and others 1983). Elevated temperature during baking would cause protein cross-links promoting loaf setting (Singh 2005). Therefore, increased cross-links enable protein molecules to aggregate closely. When the molecular size of the glutenin aggregated increases, the extractability of glutenin decreases (Lagrain and others 2005). Stathopoulos and others (2008) found that the functionality of gluten decreased during baking, and the solubility and extractability of gluten decreased rapidly between 70 and 90 °C. Surface hydrophobicity of gluten is initiated from 45 °C, indicating the exposure of hydrophobic groups of unfolded gluten polymers and consequently their decreased solubility. LMW and HMW glutenins and gliadins have different thermal stability. HMW glutenins polymerize below 100 °C, and gliadins can be polymerized directly into glutenins without any intermediate steps above 120 °C (Stathopoulos and others 2008). FTIR, near infrared spectroscopy (NIRS), gel filtration, and SE-HPLC are commonly used methods to study protein loss during baking (Belton and others 1995; Khatkar and others 1995; Bangur and others 1997; Wesley and others 2001; Feeney and others 2003; Wellner and others 2005).

### Maillard and caramelization reactions

Maillard and caramelization reactions are important reactions observed in the processing of bakery products; for example, color of crust is associated with both Maillard and caramelization reaction products (Von Elbe and Schwartz 1996; Yilmaz and Toledo 2005; Ahrné and others 2007; Purlis and Salvadori 2009). The Maillard reaction is a chemical reaction between an amino acid and a reducing sugar, while caramelization is a complex group of reactions that take place when sugars are subjected to high temperatures in the absence of amino acids (Tsai and others 2009). Both Maillard and caramelization reactions can take place simultaneously and both reactions depend on temperature, water activity, and pH (Purlis and Salvadori 2009). Crust browning occurs when the baking temperature is greater than 110 °C (Mondal and Datta 2008). During baking, water is quickly removed from the dough surface, offering optimum conditions for a Maillard reaction (Kent-Jones and Amos 1967). However, once the crust is formed, water vapor flow is restricted from pores to the dough surface (Mondal and Datta 2008). Free amino groups of lysine, peptides, or proteins could react with carbonyl groups of reducing sugars, initiating Maillard reactions under the baking conditions (Yilmaz and Toledo 2005; Michalska and others 2008). At the early stage of the Maillard reaction, Amadori rearrangement products such as furosine are generated by acid hydrolysis of the Amadori rearrangement compounds (fructosyl-lysine and lactulosyl-lysine-lysine), and at later stages, florescence compounds and cross-linking products are produced. The end products of Maillard reactions are melanoidins that are responsible for browning (Michalska and others 2008). Temperature inside the dough is much lower than that of crust, but water activity is high, causing light coloration (Borrelli and others 2003). Maillard reaction is also associated with the formation of toxic compounds such as acrylamide (Ahrné and others 2007; Gökmen and others 2007). It was found that the products of these browning reactions, especially caramelization intermediates, show antioxidant capacities (Tsai and others 2009).

### Effects of incorporated dietary fibers

Attempts to add fiber into popular foods present challenges to develop products with a fiber level that meets the requirements of The *Code of Federal Regulations* (Title 21, Part 101.54), which allows “good source of fiber” and “excellent source of fiber” claims to be made for a product. Major technical challenges of incorporating fibers will be the maximum retention of functionality of added fibers in the final finished products. Therefore, the success of DF addition should be determined based on the biological and physical effects that DF may carry into the final products. Adding insoluble DF solely to baked products is limited because of its low functionality and fermentability, in comparison, soluble fibers can be fermented (by the large intestine microflora) leading to desired metabolic effects (Prasad Rao and others 2007). Water holding capacity of soluble fibers (such as pectin and galactomannan) is greater than that of cellulose (insoluble fibers) (Ajila and others 2007). Soluble fibers are normally added together with insoluble fibers to deliver the full spectrum of fiber functionality (Brennan and Cleary 2007). Some characteristics of the added DFs, such as water solubility and coarseness, influenced the microbial ecology of the human colon and consequently digestion (Van Soes 1984). Both undigested and fermentable fibers were important to digestive activities, with fibers of moderate fermentability are preferable.

Added fibers have been found to influence the properties of other types of baked products, and these impacts may also occur to bread systems. Fibers derived from apple, lemon, and wheat have been added to cookies to replace the wheat flour on the levels of 15%, 20%, and 30% (w/w, based on the flour used), which showed that the *in vitro* protein digestibility decreased with an elevated fiber level (Bilgiçli and others 2007). Decreased crude protein contents in muffins after the addition of apple skin powder on the levels of 4%, 8%, 16%, 24%, and 32% (w/w) to replace flour also suggested a dilution effect on wheat proteins (Rupasinghe and others 2008). More interestingly, the addition of natural apple fiber (a mixture of soluble and insoluble fibers prepared using an aqueous method) into a snack bar recipe to replace 2.7% of the quick-cook rolled oats) appeared to facilitate higher contents of beneficial bioactive components including phenolics, pectic polysaccharides, and total DF (Sun-Waterhouse and others 2010). Also, the presence of these fibers (either inulin or apple DF) in the snack bar base had caused a reduced yellowness in color of the bar filling (Sun-Waterhouse and others 2010).

The physical properties of fiber including water holding, oil holding, and swelling capacity, viscosity or gel formation significantly affect product processing and quality (Collar and others 2007; Vergara-Valencia and others 2007). The addition of fibers to dough would alter dough's water absorption, causing reduced water content in dough and poor viscoelastic property. The addition of fiber may or may not decrease dough stability. Decreased dough stability and prolonged dough development time (from 4.2 to 5.8 min) were possible after the use of mango peel powder (rich in pectins) to replace flour at a level of 10% (w/w) (Ajila and others 2007). However, Wang and others (2002b) found that the added carob and pea (at the level of 3% w/w) did not alter dough development time or stability (except for the added inulin exhibiting an increasing effect). Different forms and levels of hazelnut (fine or coarse, dry or hydrated, 5% or 10% w/w) were added to bread dough, but only the added fine hazelnut powder at the level of 10% increased dough stability remarkably (Anil 2007). Thus, the type of fiber determined its impact on dough stability, possibly due to the number of hydroxyl groups of fiber that interact with water through hydrogen bonding (Wang and others 2002b).

**Figure 6:**
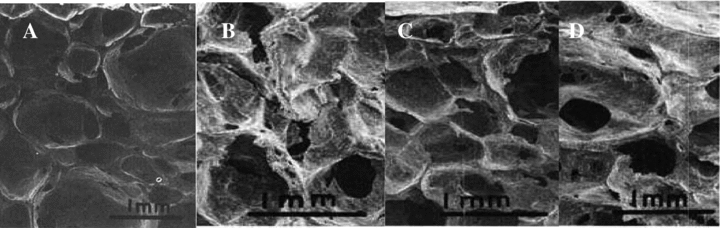
SEM micrographs of control and 15% flour replaced by fibers bread crumbs, A = control, B = cellulose, C = finely ground oat hulls, D = finely ground fine wheat bran (Pomeranz and others 1977).

The apparent negative effects of incorporated fibers on the final bread quality include reduced loaf volume, increased crumb firmness, darkened crumb appearance, and possibly tastes (Wang and others 2002b; Sangnark and Noomhorm 2004; Sudha and others 2007a, 2007b). The incorporation of apple fiber into bread might increase product density as a result of the water-binding capacity of fiber (Sudha and others 2007a). Bread volume was reduced after the addition of hazelnut fiber (Anil 2007), sugar beet fiber (Filipovic and others 2007), and apple fiber (Chen and others 1988). This phenomenon was possibly a result of the fiber weakening or crippling dough structure and reducing CO_2_ gas retention (Chen and others 1988; Sangnark and Noomhorm 2004). Moreover, appreciable amounts of water could have strongly bound to the added fibers during breadmaking, so less water was available for the development of the starch-gluten network, causing an underdeveloped gluten network and reduced loaf volume (Brennan and Cleary 2007). Therefore, the 2 mechanisms causing reduced loaf volume are the dilution of gluten, and the interactions among fiber components, water and gluten (Anil 2007). The dilution of gluten is evident by electron microscopy (Figure 6), cell walls of control bread showing a fine structure composed of numerous thin filaments connecting to adjacent cell (6A), and a reduction in fine structure in fibrous added breads, the bread crumb filaments and sheets are coarse and massive (Pomeranz and others 1977). Bread hardening effects observed after the addition of fibers results from the dilution of gluten content (Collar and others 2007). The increased dough development time and decreased dough stability caused by added apple fiber were possibly associated with slowed water hydration rate and gluten development due to increased fiber content. Increased mixing tolerance and extension value may be possible, due to interactions between fibrous materials and gluten (Sudha and others 2007b). The disruption of breadcrumb structure is due to the impairment in gas retention. Fiber addition caused shortened and low resistance to dough extension, and increased concentration of insoluble and soluble cell wall materials have been shown to partially disrupt the gluten network (Collar and others 2007).

### Effects of added phenolic antioxidants

**Figure 7:**
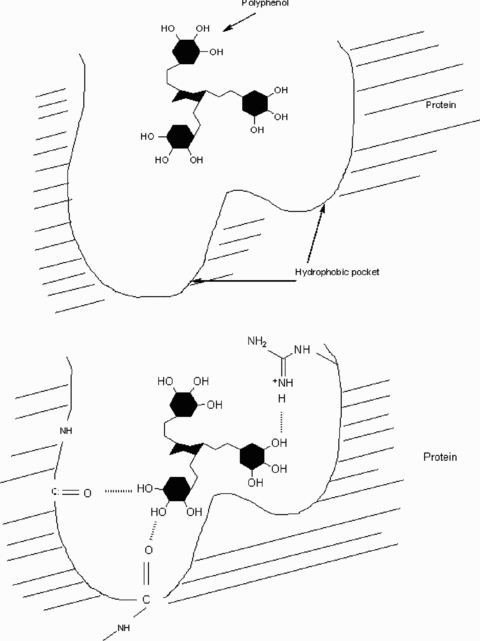
Polyphenol-protein complex: up—docking of polyphenol, down—hydrogen bonding to protein surface (adapted from Shahidi and Naczk 1995).

Phenolic antioxidants are one of the major antioxidants in wheat and can form complexes with proteins and/or polysaccharides. Such a complexation can occur reversibly via hydrogen bonding between hydroxyl groups of phenols and the carbonyl group of peptide residue of proteins (Shahidi and Naczk 1995; Renard and others 2001; Almajano and others 2007). The resultant complexes can be further stabilized via other types of bonds such as the covalent bonds and ionic bonds between phenolate and anion or cationic site of protein molecules. Hydrophobic interactions can be another means of complexation, by which polyphenol molecule attach on to the protein surface, or cross-link with different protein molecules. Figure 7 shows one type of the hydrophobic interactions between the aromatic ring of tannins and the hydrophobic region of proteins (Shahidi and Naczk 1995). The formation of complexes between phenolic antioxidants and polysaccharides is similar to that between phenolic antioxidants and proteins that is mediated by H-bonding and hydrophobic interactions. The hydrophobic interactions are facilitated by hydrophobic cavities. The resultant affinity is influenced by the molecular size, conformational flexibility of phenolic antioxidants, and water solubility of phenols (Renard and others 2001).

Processing including heating may alter the phenolic antioxidants in foods to different extents and in different ways. Previous study on ultra high temperature (UHT) milk drink containing high level of added phenolic antioxidants has shown that the stability of phenolic antioxidants could be influenced by production process and storage (Wegrzyn and others 2008). In the case of baking, there was an increase in the phenolic content of biscuits when mango peel powder was incorporated (Ajila and others 2007), whereas no change in total phenolic content was detected when apple or lemon fiber was added to cookies (Bilgiçli and others 2007). Moreover, the addition of different levels of apple skin powder had led to a range of incorporated phenolics in muffin such as flavonols, dihydrochalcones, phenolic acids, cyanidin-3-*O*-galactoside, and flavan-3-ols, and the baking process affected the level and composition of phenolics in baked muffin (for example, cyanidin-3-*O*-galactoside was the most affected phenolic compound with a 15.7% recovery) (Rupasinghe and others 2008). After a green tea extract (rich in catechins) was added to the bread dough on the levels of 50, 100, and 150 mg per 100 g flour, varied stability was detected among the individual phenolics and 83% to 91% of the total phenolics were retained (Wang and Zhou 2004). The chemical structure of some phenolics is shown in Figure 5.

Several mechanisms may be involved underlying the varied behaviors of phenolic compounds upon food processing. While the loss of catechins could be a result of the combined effect of oxidation, isomerization/epimerization, and degradation during breadmaking, the reduction of phenolic content could possibly be associated with the interactions between phenolic antioxidants and wheat proteins via hydrogen bonding during dough preparation (Wang and Zhou 2004). The detected antioxidant composition or capacity of a final baked product would derive from the intrinsic phenolic compounds of flour, added phenolic ingredients, other ingredients naturally containing phenolics, intermediate phenolic products newly generated during baking (for example, via Maillard reactions) (Michalska and others 2008), thermal-induced degradative products (Rupasinghe and others 2008), and/or polyphenol-polysaccharides complexes (Shahidi and Naczk 1995).

It is worth noting that the use of high level of added phenolic antioxidants in food formulations may lead to negative effects on the sensory attributes of finished foods such as increased bitterness and astringency (Jaeger and others 2009). However, if the level of added phenolic antioxidants is well monitored, especially when the phenolic antioxidants co-exist naturally with other active compounds such as pectic polysaccharides, the consumer acceptability of the finished food might increase due to the beneficial effects on sensory attributes derived from the interactions among phenolic antioxidants and other compounds (Sun-Waterhouse and others 2009).

### Changes in rheological properties of dough

Rheological properties of dough are very important indices for product development in terms of product quality and process efficiency (Mondal and Datta 2008). Rheology concerns the flow and deformation of a material (Vergnes 2003). The various approaches and understandings of the molecular basis of dough rheology have been well discussed and reviewed (Dobraszczyk and Morgenstern 2003; Dobraszczyk 2004; Belton 2005; Piteira and others 2006; Song and Zheng 2007; Ng and McKinley 2008). The rheological properties of gluten particularly elasticity are associated with texture, shape, and expansion of a finished product (Abang Zaidel and others 2008; Dobraszczyk and Salmanowicz 2008). There is a strong relationship between rheological properties such as viscoelasticity and the amount of HMW gluten polymers (Lefebvre and others 2000). Gluten polymer network has flexible or semiflexible chains between junction gaps of 20 nm (Ng and McKinley 2008).

Molecular weight and structure of gluten polymers are closely linked to their rheological behaviors and, ultimately, their performance in the end product (Gélinas and McKinnon 2004). Polymer characterization techniques that require solubility in water or solvent such as SE-HPLC are unable to examine gluten polymers with little solubility in water or solvent (Dobraszczyk 2004). The problems recognized in rheological studies include the measurements are not carried out in a region of extension that is appropriate to the actual mixing processes (Dobraszczyk and Morgenstern 2003). Another way is to extract the material from dough during mixing and relate the properties to the mechanical changes occurring during mixing (Belton 2005).

There are several ways to evaluate the rheological behaviors of a baked product, with *G*′ (elastic modulus), *G*″ (viscous modulus), η′ (dynamic viscosity), η″ (complex viscosity), and loss tangent tan δ (*G*″/*G*′=η′/η″) being the commonly used terms. *G*′ and *G*″ of glutens show significant positive correlations with loaf volume, and the delta value is helpful to understand behavior of a material (Steffe 1992). If a material is an ideal elastic material, the stress and strain are in phase and δ= 0. Therefore, *G*″ and η′ are also equal to 0 because there is no viscous dissipation energy (Steffe 1992; Miller and Hoseney 1999; Belton 2005). The term strength is used to describe the type of flour, and the terms “strong flour” and “weak flour” indicate flour from hard and soft wheats, respectively. Strong flours are preferred for breadmaking and weak flours for cakes and biscuits. Strong flour has a higher proportion of protein with its gluten having a good elasticity. Dough made from good quality (strong) flour has lower tan δ values than that from poor quality (weak) flour (Kokelaar and others 1996; Miller and Hoseney 1999). Soluble fractions in dough play important roles in breadmaking, and less tan δ and greater *G*′ may occur in the absence of the water-soluble fractions (Faubion and Hoseney 1997; Rouillé and others 2005). Addition of pentosan could increase tan δ and reduce *G*′ (Baltsavias and others 1997). Hydrocolloids have been incorporated in dough imparting different textural impacts on bread, for example, alginate enhances dough strength and κ-carrageenan reduces breadcrumb firmness (Rosell and others 2001). The tan δ values of glutens are ranked in the decreasing order as weak gluten > strong > extra strong glutens, while *G*′ and *G*″ values show the reverse trend (Song and Zheng 2007). The high tan δ value of doughs that were made from poor quality flour could be a result of fewer entanglements or entanglements that were easily dissociated (due to hydrophilic interaction between the gluten proteins) (Miller and Hoseney 1999). Attenburrow and others (1990) reported that *G*′ value for gluten varies from 2000 to 8000 Pa, depending on the temperature used. *G*′ increased at higher temperatures (for example, >60 °C), possibly due to gelatinization of residual starch (Kim and Cornillon 2001), and/or formation of new cross-links via disulphide bond formation (Attenburrow and others 1990). Therefore, gluten rich in HMW subunits show low *G*′ and *G*″ (Dobraszczyk and Schofield 2003). For glutens rich in HMW subunits, *G*′ and *G*″ show a slight frequency dependency and heat treatment does not significantly influence the rheological behavior. Gluten rich in LMW subunits is viscous before baking, while heat treatment might completely change it into an elastic material (Song and Zheng 2007). Gliadins can act as plasticizers, and an elevated gliadin content in gluten would result in a decrease in elasticity (Popineau and others 1994).

## Conclusions

This particular review brings to light the recent interests in nutrition and disease prevention that may drive a consumer demand for functional bread with enhanced fiber and phenolic antioxidant contents. A review of the published literature revealed that (1) breadmaking may alter the protein structure; (2) the addition of different types of fibers may affect nutritional values, antioxidant status, rheological properties, and sensory attributes of baked products; (3) baking may influence added phenolic antioxidants in free forms or as components of added ingredients; (4) research approaches of previous studies are mainly include the measurement of color, volume expansion, moisture content, fiber content, and phenolic content of the finished baked products; (5) different fibers have been incorporated into baked products, and the fibers were in pure form such as pectin or a complex form with other macro-/micro-food components such as bound phenolic antioxidants; (6) the phenolic antioxidants included in the incorporated ingredients were at relatively low level.

As pointed out throughout the text of this article, there are challenges of investigating deeper into the process of breadmaking. For example, when new bread formulations like those containing high levels of fiber polysaccharides and phenolic antioxidants are demanded, the correlation between individual phenolic compounds as a function of structure needs elucidation. The “order of addition” may be another important concern for future work when both polyphenol concentrate and fiber ingredients are required to be added into dough. The change in the “order of addition” may result in different interactive mechanisms and reaction kinetics among the dough components including wheat proteins, fiber polysaccharides, and phenolic antioxidants, which consequently alter the dough properties and bread attributes. Therefore, in order to answer the fundamental questions on the bread-improving mechanisms, future research should focus on modes of action of different ingredients and/or additives including those newly required for addition. Examination of the molecular interactions among the key dough components, and the resultant changes in polymeric network and bulk characteristics of dough and bread is required.
